# Nasopharyngeal carcinoma burden in the United States and worldwide, 1990 to 2023: A pairwise analysis with mortality projections to 2050

**DOI:** 10.1097/MD.0000000000051132

**Published:** 2026-10-16

**Authors:** Ibraheem Altamimi, Zainab Yunus Alfallatah, Abdulrahman Alzmmam, Mesfer Alsaqoor, Mohammed Ibrahim A. Almakrami, Abdulaziz Abdulmalik Althubyani, Mubarak Mohammed Alshahrani, Abdulmalik Abdulelah Bin Kassim, Mohammed Abdullah Alharbi, Muath Hamad Alqesair, Mohammed Adel Albalawi, Aalaa Saleh, Saad Hamdi Alenzi

**Affiliations:** a Dermatology Department, King Abdulaziz Medical City, Riyadh, Saudi Arabia; b Ministry of Health, Saudi Arabia; c College of Medicine, Qassim University, Qassim, Saudi Arabia; d Royal College of Surgeons in Ireland, Dublin, Ireland; e Najran University, Najran City, Saudi Arabia; f College of Medicine and Surgery, Taif University, Taif, Saudi Arabia; g Batterjee Medical College, Aseer, Saudi Arabia; h College of Medicine, Imam Mohammad Ibn Saud Islamic University (IMSIU), Riyadh, Saudi Arabia; i College of Medicine, Prince Sattam bin Abdulaziz University, Al-Kharj, Saudi Arabia; j University of Szeged Albert Szent-Györgyi, Szeged, Hungary; k Arabian Gulf University, Bahrain; l Faculty of Medicine, Lebanese University, Beirut, Lebanon; m King Fahad Specialist Hospital, Tabuk Health Cluster, Tabuk, Saudi Arabia.

**Keywords:** global, Global Burden of Disease, incidence, mortality trends, nasopharyngeal carcinoma, sex disparities, United States

## Abstract

Nasopharyngeal carcinoma (NPC) is a rare but geographically concentrated malignancy. Long-term comparative analyses of NPC trends between the United States and global populations, particularly by sex, are limited. Using Global Burden of Disease 2023 data, we analyzed temporal trends in NPC incidence, prevalence, and mortality from 1990 to 2023 in the United States and worldwide. Age-standardized rates per 100,000 population were calculated. Joinpoint regression estimated annual percent changes (APCs) and average annual percent change (AAPC) with 95% confidence intervals. Pairwise comparisons assessed differences between United States and global trends. Mortality forecasts through 2050 were generated using the established Global Burden of Disease forecasting framework. From 1990 to 2023, US age-standardized mortality declined by 44%, from 0.34 to 0.19 per 100,000 (AAPC: –1.70), while global mortality decreased from 1.46 to 0.83 (AAPC: –1.70). Global mortality increased between 2020 and 2023 (APC: 2.06), unlike the US decline. The incidence rate declined in the United States over the study period (AAPC: –1.03), while remaining largely stable globally. However, global incidence increased between 2020 and 2023 (APC: 3.86). Similarly, prevalence decreased in the United States (AAPC: –0.81) while showing a rising trend worldwide (AAPC: 0.74). Males consistently exhibited higher rates across all measures. Pairwise comparisons showed significant differences between United States and global trends. From 2023 to 2050, global NPC age-standardized mortality rate is projected to increase by 12.05% (0.93, 95% uncertainty interval [UI]: 0.75–1.16), driven by a 21.8% rise in males (1.45, 95% UI: 1.18–1.78), while declining by 11.76% in females (0.45, 95% UI: 0.33–0.63). In the United States, age-standardized mortality rate is projected to increase by 10.5% (0.21, 95% UI: 0.18–0.23), with a 17.9% rise in males (0.33, 95% UI: 0.29–0.37) and an 11.82% decline in females (0.097, 95% UI: 0.08–0.12). US NPC burden has declined steadily, while global patterns show rising prevalence and recent mortality increases. The observed differences in burden and temporal trends underscore the importance of further research into strategies for surveillance, early detection, and prevention in high-risk populations.

## 1. Introduction

Nasopharyngeal carcinoma (NPC) is a malignancy of the nasopharynx with an uneven global distribution.^[1]^ It arises from the nasopharyngeal epithelium and is classified into 3 main pathological types: squamous keratinizing, squamous nonkeratinizing, and basaloid squamous cell carcinoma.^[2]^ Nonkeratinizing carcinoma constitutes the majority of cases in high-incidence areas, while keratinizing carcinoma is the predominant form in regions with low incidence.^[3,4]^ The incidence and mortality of NPC are notably higher among populations in North Africa, the Middle East, China, and Southeast Asia.^[1]^ A 2-decade study conducted in the United States reported a stable mortality trend for NPC, accounting for approximately 14,000 deaths, with the highest disease burden observed among males, Asians, and older adults.^[5]^ Within the broader Asian demographic, Laotians and Chinese Americans represent the subgroups most affected by NPC.

Smoking, alcohol use, and preserved food consumption have been associated with NPC risk, with dietary habits potentially contributing to its high incidence among Chinese migrants.^[6]^ Infectious factors, including human papillomavirus and Epstein–Barr virus, have also been implicated in NPC pathogenesis.^[7–11]^ Men are over 3 times more likely to be diagnosed with the disease. Diagnosis most commonly occurs around 55 years, with half of the cases identified by this age.^[1,12]^ These findings may reflect greater occupational exposures among men, which often decline after age 55, as well as tobacco use and genetic susceptibility.^[13]^

Global and US research on the burden of NPC remains limited, with most studies concentrating on specific populations or regions, relying mainly on pre-2020 or older Global Burden of Disease (GBD) data, and offering minimal analysis of sex-specific trends.^[3,5,13]^ To address existing knowledge gaps, this study aimed to examine sex-specific incidence, prevalence, and mortality trends in NPC in the United States and worldwide from 1990 to 2023, and to forecast sex-specific mortality through 2050.

## 2. Methods

### 2.1. Overview

The GBD 2023 study, an expanded update of GBD 2021, provides systematic estimates for 204 countries and territories. It includes data on 292 causes of death, covering incidence, prevalence, mortality, years of life lost, and disability-adjusted life-years, and incorporates 11,474 new data sources compared with GBD 2021.^[14]^ All data were compiled by the Institute for Health Metrics and Evaluation, and the estimates adhere to the Guidelines for Accurate and Transparent Health Estimates Reporting.^[15]^

### 2.2. Protocol approval and patient consent

Data for this study were obtained from the Global Health Data Exchange query tool (http://ghdx.healthdata.org/gbd-results-tool), a publicly available, deidentified database. As all data were aggregated and anonymized, institutional review board approval and informed consent were not required.

### 2.3. Disease definition

NPC is a cancer arising from the epithelial mucosa of the nasopharynx. Within the GBD framework, causes are organized into 4 hierarchical levels, ranging from broad categories (level 1; such as nutritional deficiencies or injuries) to more specific conditions (level 4; such as hemorrhagic stroke). NPC is classified as a level 3 cause under the level 2 category of neoplasms. According to the International Classification of Diseases, 10th Revision, NPC corresponds to codes C11–C11.9 and D10.6, while in the International Classification of Diseases, 9th Revision it is coded as 147 to 147.9 and 210.9.^[14]^

### 2.4. Data sources and extraction

GBD data are compiled from a systematic review of vital registration systems, demographic censuses, and disease registries. In the United States, primary data sources included health insurance claims and encounter databases, the National Hospital Discharge Survey, the National Vital Statistics System mortality files, and the Statewide Inpatient Databases. Data for NPC were extracted for mortality, incidence, and prevalence using rate and number metrics, with age-standardized estimates reported for overall population and by sex for the United States and globally from 1990 to 2023. Data were downloaded directly from the GBD Results Tool on October 12, 2025. GBD rates are age-standardized according to the GBD standard population, as described in previous literature.^[14]^

### 2.5. Statistical analysis

Age-standardized incidence (ASIR), prevalence, and mortality rates for NPC were extracted, with all rates expressed per 100,000 population. The 95% uncertainty intervals (UIs) were derived from the 2.5th and 97.5th percentiles of 1000 ordered draws. Annual percentage changes (APCs) in ASIR, age-standardized prevalence (ASPR), and age-standardized mortality (ASMR) were estimated using Joinpoint Regression Program version 5.0.2 (National Cancer Institute).^[16]^ Log-transformed rates were modeled using the grid-search method, with the optimal number of joinpoints determined sequentially using Monte Carlo permutation tests based on 4499 permutations at an overall significance level of 0.05.^[17]^ The analysis started with 0 joinpoints and allowed the software to select the optimal number of joinpoints based on statistical significance. The minimum number of years between joinpoints was set according to the software’s default specification, that is, 2 APCs were estimated for each trend segment, and the average annual percentage change (AAPC) was calculated as a weighted average of the segment-specific APCs, with weights proportional to the length of each segment. Pairwise comparisons of AAPCs were performed using the Joinpoint regression software to assess whether the AAPCs differed significantly between the US and global series. Because the US population is included within the global estimates, the 2 series are not fully statistically independent. Therefore, the results of these pairwise comparisons were interpreted cautiously, recognizing that the overlap between the series may affect the underlying statistical assumptions and the significance of between-series differences. For mortality forecasting, the GBD framework applies a multistage modeling approach in which projected sociodemographic factors and risk exposures are incorporated into cause-specific mortality models. Mortality estimates are generated using regression-based models that account for relevant risk factor effects and temporal patterns, with forecasts produced across sexes through 2050. Further methodological details of the GBD forecasting framework have been described previously in the literature.^[18]^ Statistical significance was set at *P* < .05.

## 3. Results

### 3.1. Mortality

Between 1990 and 2023, the mortality rate related to NPC decreased in the United States and worldwide. In the United States, the ASMR declined from 0.34 (95% UI: 0.28–0.38) per 100,000 in 1990 to 0.19 (95% UI: 0.17–0.22) per 100,000 in 2023, concurrent with minimal increase in the absolute deaths from 991 (95% UI: 823–1129) in 1990 to 1033 (95% UI: 890–1206) in 2023 (Table 1). Regarding sex, the male mortality rate decreased from 0.5 (0.4–0.6) to 0.28 (0.23–0.34); likewise, the mortality rate among females decreased as well from 0.2 (95% UI: 0.16–0.25) to 0.11 (95% UI: 0.08–0.13). Globally, mortality demonstrated a decreasing trend as well, with the ASMR declining from 1.46 (95% UI: 1.23–1.66) in 1990 to 0.83 (95% UI: 0.7–0.99) in 2023. The global absolute number of deaths demonstrated a minimal increase as well from 62,829 (95% UI: 52,753–71,572) to 75,395 (95% UI: 63,292–89,144), indicating global growth as well. In regard to sex, mortality rates are higher in males than females: 2.07 (95% UI: 1.75–2.44) versus 0.91 (95% UI: 0.56–1.13) in 1990 and 1.19 (95% UI: 0.95–1.46) versus 0.51 (95% UI: 0.38–0.66) in 2023.

**Table 1:** Age-standardized mortality, incidence, and prevalence rates attributed to nasopharyngeal carcinoma in the United States and worldwide; comparison of 1990 and 2023.

Variable	1990						2023							
	Incidence		Prevalence		Mortality		Incidence		Prevalence		Mortality			
	Cases	Age-adjusted rate	Cases	Age-adjusted rate*	Cases	Age-adjusted rate*	Cases	Age-adjusted rate*	Cases	Age-adjusted rate*	Cases	Age-adjusted rate*		
US														
Overall	2622 (1750–3775)	0.93 (0.61–1.34)	14,510 (8642–22,407)	5.22 (3.11–8.07)	991 (823–1129)	0.34 (0.28–0.38)	3136 (2072–4550)	0.66 (0.43–0.96)	18,354 (11,155–28,239)	4.01 (2.43–6.14)	1033 (890–1206)		0.19 (0.17–0.22)	
Male	1636 (1108–2448)	1.25 (0.84–1.87)	8831 (5026 to 14,465)	6.74 (3.83–11.02)	660 (535–790)	0.5 (0.4–0.6)	2116 (1315–3303)	0.91 (0.55–1.42)	12,214 (6807–20,688)	5.4 (2.97–9.11)	725 (600–879)		0.28 (0.23–0.34)	
Female	986 (637–1434)	0.65 (0.42–0.95)	5679 (3505 to 8755)	3.88 (2.39–5.96)	331 (261–408)	0.2 (0.16–0.25)	1020 (701–1472)	0.43 (0.29–0.62)	6140 (3890–9105)	2.69 (1.71–3.98)	308 (239–383)		0.11 (0.08–0.13)	
Global														
Overall	89,353 (73,918–108,099)	2.01 (1.67–2.4)	356,487 (277,531–481,168)	7.76 (6.1–10.39)	62,830 (52,753–71,572)	1.46 (1.23–1.66)	169,209 (130,838–221,494)	1.89 (1.46–2.49)	875,774 (634,408–1,210,740)	9.84 (7.11–13.6)	75,395 (63,293–89,144)			0.83 (0.7–0.99)
Male	58,312 (47,761 to 71,147)	2.73 (2.26–3.31)	225,386 (172,337 to 306,789)	10.11 (7.77–13.66)	42,479 (35,892–50,039)	2.07 (1.75–2.44)	115,140 (89,459–153,967)	2.64 (2.05–3.53)	592,066 (433,751–838,696)	13.54 (9.92–19.22)	52,030 (41,781–64,026)			1.19 (0.95–1.46)
Female	31,041 (19,107 to 40,294)	1.34 (0.83–1.74)	131,101 (81,166 to 181,702)	5.54 (3.48–7.61)	20,351 (12,443–25,482)	0.91 (0.56–1.13)	54,070 (37,553–74,945)	1.2 (0.83–1.66)	283,708 (185,353–413,987)	6.34 (4.15–9.27)	23,365 (17,775–30,255)			0.51 (0.38–0.66)

US = United States of America.

* All reported rates are expressed per 100,000 population with uncertainty intervals.

In the United States, mortality rates for NPC showed a significant overall decline, with an AAPC of −1.70% (95% confidence interval [CI]: −1.89 to −1.51) (Table 2, Fig. 1) This reduction was most pronounced from 1995 to 2004 (APC: −2.62*, 95% CI: −3.05 to −2.19), followed by a more moderate but sustained decrease from 2004 to 2023 (APC: −1.39*, 95% CI: −1.52 to −1.27). This trend was observed in both females (AAPC: −1.89*, 95% CI: −2.45 to −1.33) and males (AAPC: −1.70*, 95% CI: −1.83 to −1.57), with both sexes experiencing the sharpest declines between 1995 and the early 2000s. On a global scale, a similar significant reduction in mortality was observed (AAPC: −1.70*, 95% CI: −1.92 to −1.48). The global trend was characterized by a particularly steep decrease from 1999 to 2005 (APC: −4.47*, 95% CI: −5.04 to −3.90). However, this long-term decline has reversed in recent years, with a significant increase in mortality observed from 2020 to 2023 (APC: 2.06*, 95% CI: 0.29–3.86). This recent reversal was evident in both females (APC: 3.93*, 95% CI: 2.64–5.24) and males (APC: 2.03*, 95% CI: 0.16–3.94) (Figures S1 and S2, Supplemental Digital Content 1).

**Table 2:** Mortality, incidence and prevalence rates related to nasopharyngeal carcinoma in the United States and worldwide from 1990 to 2023.

Variable		Trend segment	Start point	End point	Annual percentage change	Average annual percentage change	*P* value
US							
Mortality	Overall	1	1990	1995	−1.22* (−2.16 to −0.27)	−1.70* (−1.89 to −1.51)	<.000001
		2	1995	2004	−2.62* (−3.05 to −2.19)		
		3	2004	2023	−1.39* (−1.52 to −1.27)		
	Female	1	1990	2008	−2.15* (−2.34 to −1.96)	−1.89* (−2.45 to −1.33)	<.000001
		2	2008	2011	−3.72 (−9.62 to 2.58)		
		3	2011	2023	−1.04* (−1.40 to −0.68)		
	Male	1	1990	1995	−1.15* (−1.77 to −0.53)	−1.70* (−1.83 to −1.57)	<.000001
		2	1995	2003	−2.77* (−3.14 to −2.39)		
		3	2003	2023	−1.41* (−1.49 to −1.34)		
Incidence	Overall	1	1990	1998	−0.01 (−0.31 to 0.29)	−1.03* (−1.15 to −0.90)	<0.000001
		2	1998	2019	−1.65* (−1.72 to −1.58)		
		3	2019	2023	0.21 (−0.61 to 1.05)		
	Female	1	1990	1997	−0.66* (−1.09 to −0.22)	−1.23* (−1.52 to −0.94)	<.000001
		2	1997	2009	−1.61* (−1.80 to −1.41)		
		3	2009	2012	−3.37* (−5.87 to −0.79)		
		4	2012	2018	−1.67* (−2.34 to −1.00)		
		5	2018	2023	0.72 (−0.00 to 1.45)		
	Male	1	1990	1995	0.69* (0.30–1.09)	−0.94* (−1.15 to −0.73)	<.000001
		2	1995	2000	−0.48 (−1.05 to 0.09)		
		3	2000	2003	−2.66* (−4.45 to −0.84)		
		4	2003	2010	−0.95* (−1.26 to −0.65)		
		5	2010	2019	−1.92* (−2.11 to −1.74)		
		6	2019	2023	0.02 (−0.54 to 0.58)		
Prevalence	Overall	1	1990	1999	0.48* (0.27–0.69)	−0.81* (−0.99 to −0.63)	<.000001
		2	1999	2004	−2.00* (−2.71 to −1.29)		
		3	2004	2009	−0.87* (−1.59 to −0.14)		
		4	2009	2019	−2.00* (−2.19 to −1.81)		
		5	2019	2023	0.89* (0.21–1.57)		
	Female	1	1990	1999	−0.57* (−0.85 to −0.29)	−1.10* (−1.33 to −0.86)	<.000001
		2	1999	2009	−1.62* (−1.88 to −1.36)		
		3	2009	2013	−2.94* (−4.21 to −1.65)		
		4	2013	2019	−1.44* (−2.09 to -0.80)		
		5	2019	2023	1.44* (0.50–2.39)		
	Male	1	1990	1993	1.79* (0.87–2.73)	−0.66* (−0.83 to −0.50)	<.000001
		2	1993	1999	0.72* (0.30–1.14)		
		3	1999	2004	−2.04* (−2.59 to −1.49)		
		4	2004	2010	−0.61* (−1.01 to −0.21)		
		5	2010	2019	−2.11* (−2.30 to −1.92)		
		6	2019	2023	0.41 (−0.14 to 0.97)		
Global							
Mortality	Overall	1	1990	1999	−1.83* (−2.09 to −1.57)	−1.70* (−1.92 to −1.48)	<.000001
		2	1999	2005	−4.47* (−5.04 to −3.90)		
		3	2005	2013	−1.95* (−2.27 to −1.62)		
		4	2013	2020	−0.42 (−0.88 to 0.04)		
		5	2020	2023	2.06* (0.29–3.86)		
	Female	1	1990	2000	−2.40* (−2.61 to −2.19)	−1.77* (−2.04 to −1.50)	<.000001
		2	2000	2004	−5.73* (−6.64 to −4.81)		
		3	2004	2013	−2.27* (−2.48 to −2.05)		
		4	2013	2017	0.60 (−0.36 to 1.56)		
		5	2017	2020	−1.42 (−3.53 to 0.73)		
		6	2020	2023	3.93* (2.64–5.24)		
	Male	1	1990	1999	−1.60* (−1.86 to −1.34)	−1.66* (−1.89 to −1.42)	<.000001
		2	1999	2005	−4.30* (−4.84 to −3.76)		
		3	2005	2014	−1.90* (−2.18 to −1.61)		
		4	2014	2020	−0.49 (−1.17 to 0.19)		
		5	2020	2023	2.03* (0.16–3.94)		
Incidence	Overall	1	1990	1999	−0.52* (−0.78 to −0.25)	−0.17 (−0.42 to 0.08)	.185093
		2	1999	2004	−3.40* (−4.24 to −2.55)		
		3	2004	2013	−0.29 (−0.60 to 0.03)		
		4	2013	2020	1.08* (0.58–1.59)		
		5	2020	2023	3.86* (1.89–5.87)		
	Femal**e**	1	1990	1999	−1.02* (−1.23 to −0.81)	−0.36* (−0.63 to −0.08)	.010810
		2	1999	2004	−3.84* (−4.42 to −3.26)		
		3	2004	2008	0.06 (−0.85 to 0.98)		
		4	2008	2013	−0.84* (−1.39 to −0.28)		
		5	2013	2017	1.89* (0.93–2.86)		
		6	2017	2020	0.18 (−1.85 to 2.26)		
		7	2020	2023	4.38* (3.12–5.66)		
	Male	1	1990	1999	−0.29* (−0.57 to −0.01)	−0.07 (−0.34 to 0.19)	.580540
		2	1999	2004	−3.17* (−4.05 to −2.28)		
		3	2004	2013	−0.26 (−0.60 to 0.07)		
		4	2013	2020	1.03* (0.50–1.57)		
		5	2020	2023	3.89* (1.86–5.95)		
Prevalence	Overall	1	1990	1998	0.73* (0.41–1.06)	0.74* (0.42–1.07)	0.000007
		2	1998	2004	−2.29* (−2.90 to -1.67)		
		3	2004	2007	1.88 (−0.78 to 4.60)		
		4	2007	2012	0.15 (−0.70 to 1.01)		
		5	2012	2020	1.76* (1.39–2.13)		
		6	2020	2023	4.14* (2.55–5.75)		
	Female	1	1990	1999	−0.11 (−0.32 to 0.10)	0.39* (0.14–0.63)	0.002021
		2	1999	2004	−3.07* (−3.69 to -2.45)		
		3	2004	2007	1.84 (−0.11 to 3.82)		
		4	2007	2012	0.03 (−0.60 to 0.67)		
		5	2012	2020	1.52* (1.24–1.79)		
		6	2020	2023	3.96* (2.71–5.22)		
	Male	1	1990	1998	1.08* (0.70–1.45)	0.93* (0.58–1.28)	<.000001
		2	1998	2004	−2.08* (−2.75 to −1.39)		
		3	2004	2007	2.03 (−0.76 to 4.89)		
		4	2007	2012	0.24 (−0.71 to 1.20)		
		5	2012	2020	1.86* (1.46–2.27)		
		6	2020	2023	4.24* (2.49–6.03)		

AAPC = average annual percent change, APC = annual percent change, US = United States of America.

* APC or AAPC is significantly different from 0 at the α = 0.05 level.

**Figure 1.**
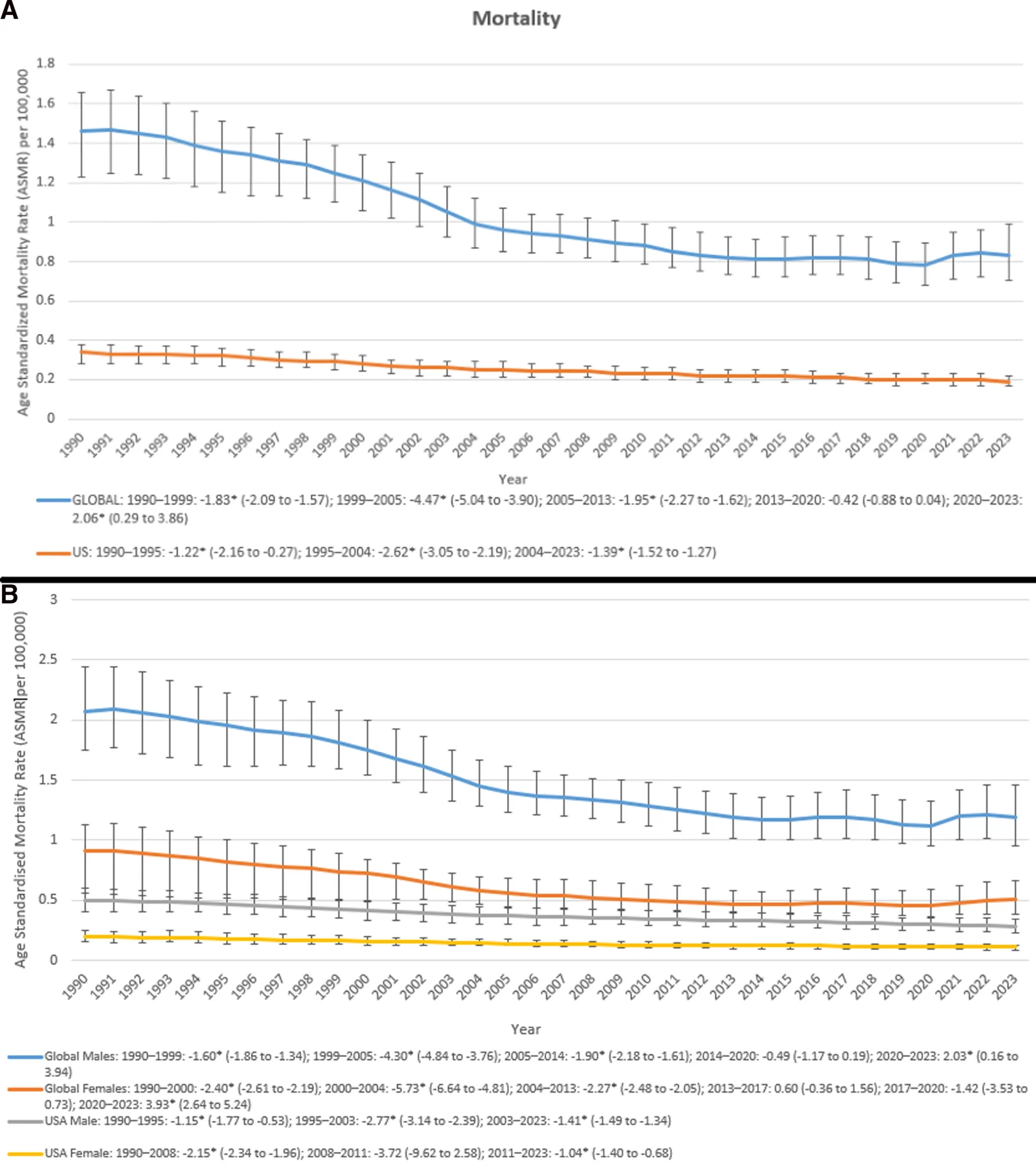
Trends in age-standardized mortality rates for nasopharyngeal carcinoma, 1990 to 2023. (A) Global and US mortality rates. (B) Mortality rates stratified by sex globally and in the United States. Error bars represent 95% uncertainty intervals. Joinpoint-derived annual percentage changes with corresponding 95% confidence intervals are provided in the legend. Rates are expressed per 100,000 population.

The trend among US males differed significantly from that of global males (*P* = .000222), and a similar disparity was observed among females (*P* < .000001). When both sexes were combined, the overall US trend also differed significantly from the global pattern (*P* = .000222) (Table 3). From 2023 to 2050, the ASMR for NPC is projected to rise by about 12.05% (0.93, 95% UI: 0.75–1.16), with a 21.8% (1.45, 95% UI: 1.18–1.78) increase among males and a 11.76% (0.45, 95% UI: 0.33–0.63) decline among females globally. In comparison, the ASMR in the United States is expected to increase by approximately 10.5% (0.21, 95% UI: 0.18–0.23), during the same period, largely due to a 17.9% (0.33, 95% UI: 0.29–0.37) rise among males, while female rates are anticipated to fall by 11.82% (0.097, 95% UI: 0.08–0.12) (Figures S3–S5, Supplemental Digital Content 2).

**Table 3:** Pairwise comparison between mortality, incidence, and prevalence rates average annual percentage changes related to nasopharyngeal carcinoma in the United States and worldwide from 1990 to 2023.

Variable	US		Variable	Global		*P* value for AAPC comparison
	AAPC (95% CI)	*P* value		AAPC (95% CI)	*P* value	
Mortality						
Overall	−1.70* (−1.89 to −1.51)	<.000001	Overall	−1.70* (−1.92 to −1.48)	<.000001	.000222
Male	−1.70* (−1.83 to −1.57)	<.000001	Male	−1.66* (−1.89 to −1.42)	<.000001	.000222
Female	−1.89* (−2.45 to −1.33)	<.000001	Female	−1.77* (−2.04 to −1.50)	<.000001	<.000001
Male	−1.70* (−1.83 to −1.57)	<.000001	Female	−1.77* (−2.04 to −1.50)	<.000001	.000222
Incidence						
Overall	−1.03* (−1.15 to −0.90)	<.000001	Overall	−0.17 (−0.42 to 0.08)	.185093	.000222
Male	−0.94* (−1.15 to −0.73)	<.000001	Male	−0.07 (−0.34 to 0.19)	.580540	.000222
Female	−1.23* (−1.52 to −0.94)	<.000001	Female	−0.36* (−0.63 to −0.08)	.010810	<.000001
Male	−0.94* (−1.15 to −0.73)	<.000001	Female	−0.36* (−0.63 to −0.08)	.010810	<.000001
Prevalence						
Overall	−0.81* (−0.99 to −0.63)	<.000001	Overall	0.74* (0.42–1.07)	.000007	<.000001
Male	−0.66* (−0.83 to −0.50)	<.000001	Male	0.93* (0.58–1.28)	<.000001	.049568
Female	−1.10* (−1.33 to −0.86)	<.000001	Female	0.39* (0.14–0.63)	.002021	<.000001
Male	−0.66* (−0.83 to −0.50)	<.000001	Female	0.39* (0.14–0.63)	.002021	.000222

AAPC = average annual percent change, CI = confidence interval, US = United States of America.

* AAPC is significantly different from 0 at the α = 0.05 level.

### 3.2. Incidence

Over the period from 1990 to 2023, the ASIR of NPC demonstrated a decreasing trend in the United States as well as globally. In the United States, the incidence rate declined from 0.93 (95% UI: 0.61–1.34) per 100,000 population in 1990 to 0.66 (95% UI: 0.43–0.96) in 2023 (Table 1). The overall number of new cases increased slightly from 2622 (95% UI: 1750–3775) to 3136 (95% UI: 2072–4550). Regarding sex differences, although both sexes exhibited a declining trend over time, males showed consistently higher incidence rates than females: 1.25 (95% UI: 0.84–1.87) versus 0.65 (95% UI: 0.42–0.95) per 100,000 in 1990, and 0.91 (95% UI: 0.55–1.42) versus 0.43 (95% UI: 0.29–0.62) per 100,000 in 2023. Globally, the ASIR declined slightly from 2.01 (95% UI: 1.67–2.40) in 1990 to 1.89 (95% UI: 1.46–2.49) in 2023, comparable to the trend in the United States, the total number of new cases also increased, almost doubled from 89,353 (95% UI: 73,918–108,099) to 169,209 (95% UI: 130,838–221,494). Similarly, males continued to demonstrate almost double the incidence rate compared to females.

In the United States, the incidence of NPC decreased significantly over the study period, with an overall AAPC of −1.03% (95% CI: −1.15 to −0.90). Following a period of stability from 1990 to 1998, the most substantial decline occurred between 1998 and 2019 (APC: −1.65, 95% CI: −1.72 to −1.58) (Table 2, Fig. 2). Females experienced a more pronounced overall reduction (AAPC: −1.23, 95% CI: −1.52 to −0.94) compared with males (AAPC: −0.94, 95% CI: −1.15 to −0.73). Notably, the male incidence trend began with a significant increase from 1990 to 1995 (APC: 0.69, 95% CI: 0.30–1.09) before embarking on a prolonged decline.

**Figure 2.**
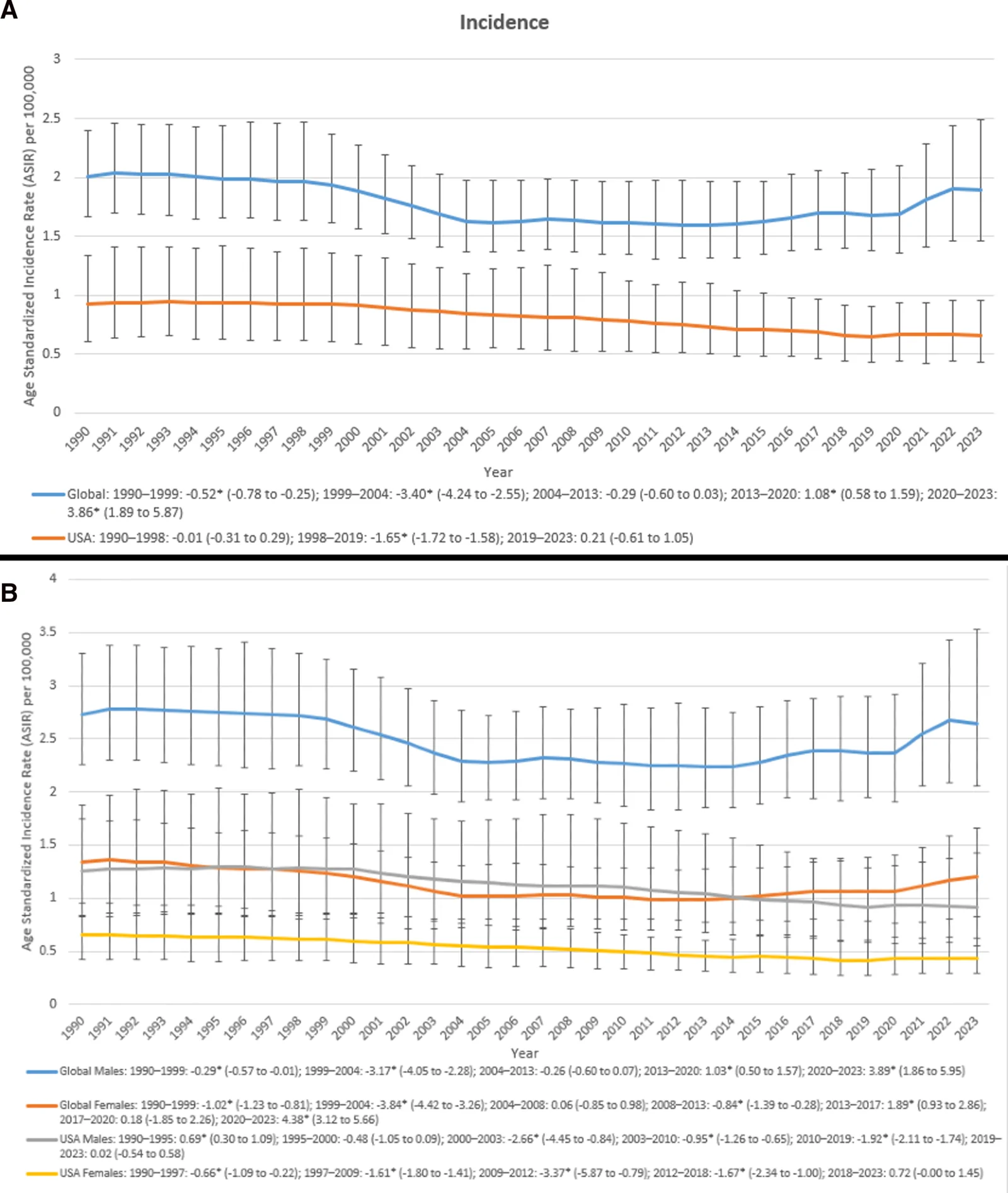
Trends in age-standardized incidence rates for nasopharyngeal carcinoma, 1990 to 2023. (A) Global and US incidence rates. (B) Incidence rates stratified by sex globally and in the United States. Error bars represent 95% uncertainty intervals. Joinpoint-derived annual percentage changes with corresponding 95% confidence intervals are provided in the legend. Rates are expressed per 100,000 population.

Conversely, the global incidence rate remained stable, showing no significant overall change from 1990 to 2023 (AAPC: -0.17, 95% CI: −0.42 to 0.08). This stability masks a dynamic pattern, including a sharp decline from 1999 to 2004 (APC: −3.40, 95% CI: −4.24 to −2.55) and a significant increase from 2013 to 2020 (APC: 1.08, 95% CI: 0.58–1.59) and 2020 to 2023 (APC: 3.86, 95% CI: 1.89–5.87). The female trend showed a slight but significant overall decrease (AAPC: −0.36*, 95% CI: −0.63 to −0.08), while the male trend remained stable with no significant overall change (AAPC: −0.07, 95% CI: −0.34 to 0.19) (Figures S6 and S7, Supplemental Digital Content 3).

The trend among US males differed significantly from that of global males (*P* = .000222), and a similar disparity was observed among females (*P* < .000001). When both sexes were combined, the overall US trend also differed significantly from the global pattern (*P* = .000222) (Table 3).

### 3.3. Prevalence

From 1990 to 2023, the prevalence of NPC in the United States, as well as worldwide, demonstrated an increase in the absolute number of prevalent cases and decreased ASPR (Table 1). In the United States, the number of prevalent cases escalated from 14,510 (95% UI: 8642–22,407) in 1990 to 18,354 (95% UI: 11,154–28,239) in 2023. Nonetheless, the ASPR declined from 5.22 (95% UI: 3.11–8.07) per 100,000 population in 1990 to 4.01 (95% UI: 2.43–6.14) per 100,000 in 2023. With respect to sex differences, males continued to exhibit higher prevalence rates compared with females: 6.74 (95% UI: 3.83–11.02) versus 3.88 (95% UI: 2.39–5.96) in 1990, and 5.40 (95% UI: 2.97–9.11) versus 2.69 (95% UI: 1.71–3.98) in 2023, despite the overall decline observed in both sexes. Globally, a distinct variation was observed, with both the absolute number of prevalent cases and the ASPR showing an upward trend. The total number of individuals living with NPC increased substantially from 356,487 (95% UI: 277,531–481,167) in 1990 to 875,774 (95% UI: 634,407–1210,740) in 2023, while the equivalent ASPR grew from 7.76 (95% UI: 6.10–10.39) to 9.84 (95% UI: 7.11–13.60) per 100,000. This reflects a rising global burden of NPC with time. With respect to sex, males maintained higher prevalence rates than females: 10.11 (95% UI: 7.77–13.66) versus 5.54 (95% UI: 3.48–7.61) per 100,000 in 1990, and 13.54 (95% UI: 9.92–19.22) versus 6.34 (95% UI: 4.15–9.27) per 100,000 in 2023.

In the United States, prevalence rates experienced a significant overall decline (AAPC: −0.81*, 95% CI: −0.99 to −0.63). This trend was characterized by an initial period of increase from 1990 to 1999 (APC: 0.48*, 95% CI: 0.27–0.69), followed by a prolonged decrease until 2019 (Table 2, Fig. 3). However, a significant upward trend has emerged recently, from 2019 to 2023 (APC: 0.89*, 95% CI: 0.21–1.57). The overall decline in prevalence was more pronounced in females (AAPC: −1.10*, 95% CI: −1.33 to −0.86) than in males (AAPC: −0.66*, 95% CI: −0.83 to −0.50). Globally, and in stark contrast to the United States, the prevalence of NPC showed a significant and sustained increase over the study period (AAPC: 0.74*, 95% CI: 0.42–1.07). This upward trend was particularly notable in recent years, with the sharpest increase occurring from 2020 to 2023 (APC: 4.14*, 95% CI: 2.55–5.75). The increase in prevalence was more pronounced in males (AAPC: 0.93*, 95% CI: 0.58 to 1.28) compared with females (AAPC: 0.39*, 95% CI: 0.14–0.63) (Figures S8 and S9, Supplemental Digital Content 4).

**Figure 3.**
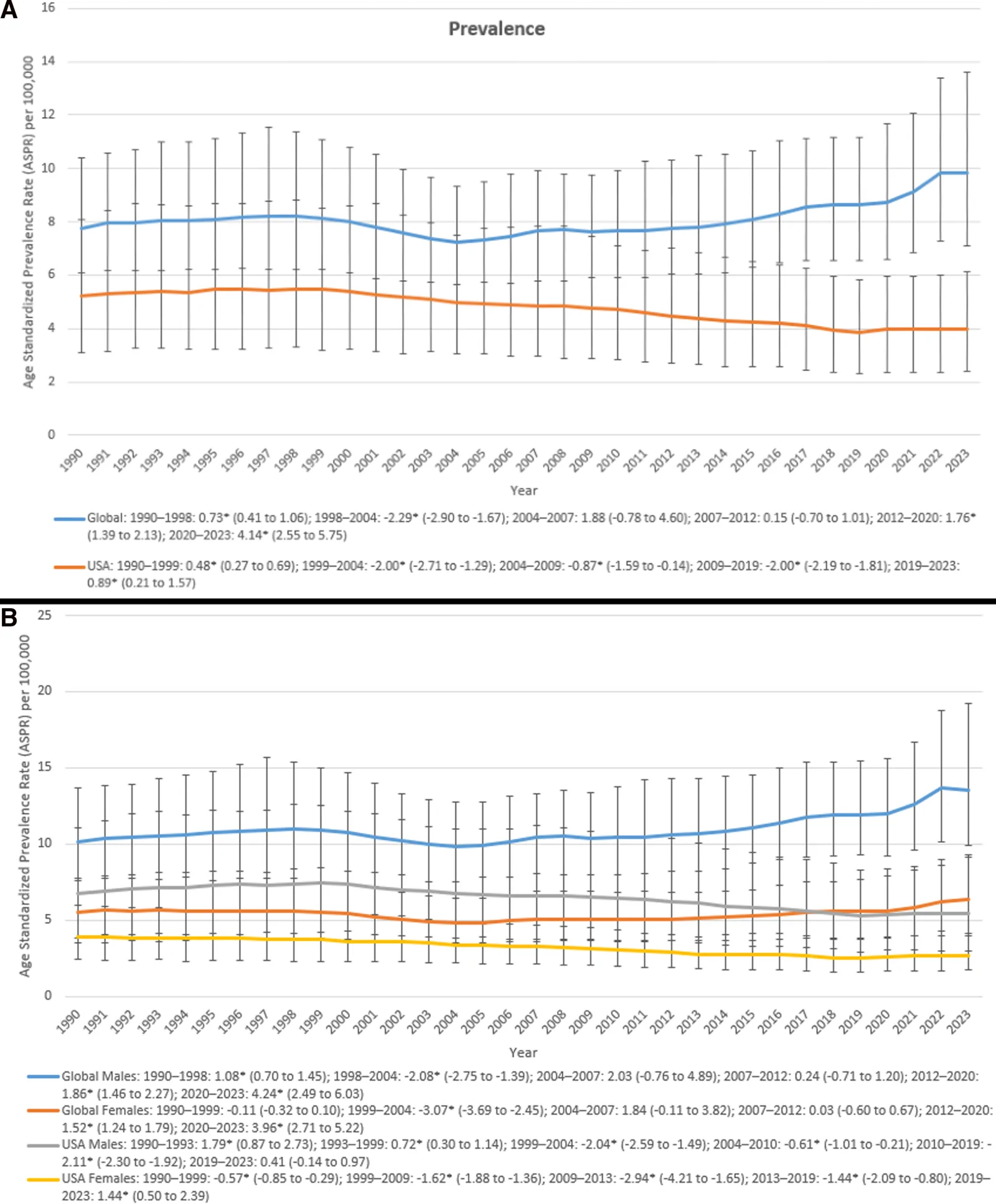
Trends in age-standardized prevalence rates for nasopharyngeal carcinoma, 1990 to 2023. (a) Global and US prevalence rates. (b) Prevalence rates stratified by sex globally and in the United States. Error bars represent 95% uncertainty intervals. Joinpoint-derived annual percentage changes with corresponding 95% confidence intervals are provided in the legend. Rates are expressed per 100,000 population.

The trend among US males differed significantly from that of global males (*P* = .049568), and a similar disparity was observed among females (*P* < .000001). When both sexes were combined, the overall US trend also differed significantly from the global pattern (*P* < .000001) (Table 3).

## 4. Discussion

Over the past 3 decades, the burden of NPC has shown distinct patterns in the United States and globally. The United States experienced sustained declines in ASIR, prevalence, and mortality, whereas global incidence remained broadly stable and mortality declined. Despite these favorable rate trends, population growth contributed to an increase in the absolute number of cases and deaths. Prevalence patterns were particularly divergent, with declining ASPR in the United States but increasing prevalence globally. The recent rise in global incidence and mortality after 2020 further highlights a departure from the longer-term global trend.

In contrast to earlier reports that described a stable NPC burden in the United States during the 20th century, our analysis reveals a consistent decline in ASIR, ASPR, and ASMR over the study period.^[19,20]^ This progressive decline in mortality may partly reflect advances in therapeutic strategies and other improvements in NPC management over time. Earlier, treatment for NPC primarily consisted of once-daily radiotherapy supplemented with a brachytherapy boost.^[21]^ Later, patients were treated with twice-daily radiotherapy through opposed lateral fields encompassing the primary site and cervical regions.^[22]^ In recent years, combination regimens incorporating gemcitabine, methotrexate, cisplatin, bleomycin, and 5-fluorouracil were introduced as concurrent or neoadjuvant chemotherapy. Integrating chemotherapy with radiotherapy has been shown to lower the incidence of distant metastases and improve disease-free survival. The decrease in NPC incidence in the United States may also be partly related to changes in environmental and lifestyle exposures reported in previous studies. For instance, the decline in smoking has been associated with a reduced incidence of type I NPC.^[23]^ Globally, the prevalence of NPC has increased, with certain regions such as China and Greenland carrying a higher burden. Dietary factors have been proposed as possible contributors to this pattern, as food samples, particularly fish from these regions, have been found to contain higher concentrations of nitrosamines compared with those from Western countries.^[24]^ Studies of migration patterns further support the possible role of environmental and lifestyle influences, as first-generation Chinese immigrants to the United States retain the high incidence rates seen in their native population, while second-generation immigrants show a gradual decline in risk as they adapt to Western lifestyles.^[25]^ In recent years, a modest global rise in NPC prevalence may partly reflect the effects of the coronavirus disease 2019 pandemic. Diagnosis and treatment delays, along with registry and case ascertainment disruptions, may have contributed to these trends.^[26,27]^ During the pandemic, outpatient visits declined sharply, and because severe acute respiratory syndrome coronavirus 2 spreads through droplets and direct contact,^[28]^ the number of endoscopic procedures was also strictly limited.^[26]^ However, the observed increase may also reflect changes in incidence, survival, population structure, detection, or GBD modeling assumptions, and cannot be attributed directly to the pandemic.

Another characteristic feature of NPC is its marked male predominance, with a male-to-female ratio typically ranging from 2- to 3-fold across different populations.^[13,29]^ In 2023, our study observed a ratio of 2.5 in the United States and 2.3 globally, consistent with previously reported findings. Several large clinical and registry-based studies have also demonstrated superior disease-specific and overall survival in females.^[30,31]^ This survival advantage is most pronounced in premenopausal women, decreases after menopause, and often disappears in older age, suggesting a possible hormonal influence underlying sex-related differences in prognosis.^[31]^

Genetic variation in NPC may be influenced by the X chromosome in males, alongside chromosome 6, which contains the well-known human leukocyte antigen susceptibility loci. X-linked effects may also contribute to sex differences in NPC incidence, although the underlying mechanisms remain unclear.^[32]^ X-chromosome inactivation, escape of some tumor suppressor genes, and sex-specific chromatin accessibility may further contribute to differences in disease susceptibility between males and and females.^[33,34]^

The more pronounced decline in NPC burden observed among both males and females in the United States, compared with global trends, may be associated with advancements in public health measures and healthcare delivery. Greater awareness of early symptoms, improved access to diagnostic services, and the widespread availability of advanced imaging and radiotherapy may have contributed to earlier detection and more effective treatment. Population-based screening in high-risk groups, combined with public education on modifiable risk factors such as smoking and dietary habits, may also be potential contributors.^[5]^ These factors represent possible explanations for the observed differences and warrant further investigation, as their individual contributions were not evaluated in the present study.

The GBD methodology has several limitations relevant to this analysis. First, because GBD data are aggregated and derived from multiple sources, variations in data quality and completeness may exist across regions and time periods. Such variability could affect the accuracy of our estimates for trends in NPC burden. Second, although the GBD employs comprehensive modeling techniques, these methods may not capture the clinical and demographic detail necessary for a deeper understanding of differences in NPC outcomes. Furthermore, as the database focuses on disease burden indicators rather than specific clinical variables, it limits the ability to fully explore the underlying determinants of observed disparities. Because global estimates aggregate countries with diverse baseline risks, data quality, and healthcare conditions, they may mask substantial regional variation and should therefore be interpreted cautiously. In addition, excluding the United States from the global comparator group is not feasible using the GBD database,^[35]^ resulting in partial overlap and limiting the statistical independence of the 2 series. This study also relied on aggregate data, which carries the risk of ecological fallacy. The use of modeled estimates introduces uncertainty, particularly in data-sparse settings, while the ecological design limits causal inference and direct attribution of temporal changes. Long-term forecasts are also subject to uncertainty. Sensitivity analyses excluding or separately modeling the pandemic period were not performed. Lastly, the GBD database does not include information on NPC-related complications, restricting a complete assessment of the disease’s overall impact across populations.

## 5. Conclusion

Between 1990 and 2023, NPC showed a consistent decline in ASMR in both the United States and globally, while ASIR declined in the United States but remained broadly stable globally, with an increase during 2020 to 2023. Despite these rate patterns, absolute case numbers increased. In contrast, prevalence demonstrated divergent trends: while ASPR declined in the United States, it rose globally. Although long-term declines were observed, recent years revealed a slight reversal in global mortality, suggesting evolving epidemiologic patterns. Projections to 2050 indicate divergent sex-specific trends in ASMR, with increases among males and declines among females in the United States and globally. Further research is needed to understand the factors underlying these trends and disparities.

## Author contributions

**Investigation:** Ibraheem Altamimi, Zainab Yunus Alfallatah, Muath Hamad Alqesair.

**Methodology:** Ibraheem Altamimi, Zainab Yunus Alfallatah, Mohammed Ibrahim A. Almakrami, Mubarak Mohammed Alshahrani, Mohammed Abdullah Alharbi, Muath Hamad Alqesair.

**Writing – original draft:** Ibraheem Altamimi, Zainab Yunus Alfallatah, Abdulrahman Alzmmam, Mesfer Alsaqoor, Mohammed Ibrahim A. Almakrami, Abdulaziz Abdulmalik Althubyani, Abdulmalik Abdulelah Bin Kassim, Mohammed Abdullah Alharbi, Muath Hamad Alqesair, Aalaa Saleh, Saad Hamdi Alenzi.

**Writing – review & editing:** Ibraheem Altamimi, Abdulrahman Alzmmam, Mesfer Alsaqoor, Mohammed Ibrahim A. Almakrami, Abdulaziz Abdulmalik Althubyani, Abdulmalik Abdulelah Bin Kassim, Mohammed Abdullah Alharbi, Muath Hamad Alqesair, Aalaa Saleh, Saad Hamdi Alenzi.

**Formal analysis:** Zainab Yunus Alfallatah, Mohammed Ibrahim A. Almakrami, Mubarak Mohammed Alshahrani.

**Funding acquisition:** Zainab Yunus Alfallatah.

**Project administration:** Zainab Yunus Alfallatah, Abdulrahman Alzmmam, Mesfer Alsaqoor, Mohammed Ibrahim A. Almakrami, Mubarak Mohammed Alshahrani, Muath Hamad Alqesair, Aalaa Saleh.

**Conceptualization:** Mesfer Alsaqoor, Aalaa Saleh.

**Supervision:** Mesfer Alsaqoor, Mohammed Adel Albalawi.

**Data curation:** Mohammed Ibrahim A. Almakrami.

**Visualization:** Abdulaziz Abdulmalik Althubyani.

**Software:** Mohammed Adel Albalawi.














